# Complications Subsequent to Urinary Tract Stent Placement: An Overview Focusing on the Imaging of Cancer Patients

**DOI:** 10.3390/medicina60020338

**Published:** 2024-02-19

**Authors:** Antonio Corvino, Luigi Basile, Giulio Cocco, Andrea Delli Pizzi, Domenico Tafuri, Fabio Corvino, Orlando Catalano

**Affiliations:** 1 Medical, Movement and Wellbeing Sciences Department, University of Naples “Parthenope”, Via Medina 40, I-80133 Naples, Italy; domenico.tafuri@uniparthenope.it; 2 Advanced Biomedical Sciences Department, University Federico II of Naples, I-80131 Naples, Italy; basileluigi92@gmail.com; 3 Department of Neuroscience, Imaging and Clinical Sciences, University “G. d’Annunzio”, I-66100 Chieti, Italy; cocco.giulio@gmail.com; 4 Departiment of Innovative Technologies in Medicine and Dentistry, University “G. d’Annunzio”, I-66100 Chieti, Italy; andreadellipizzi@gmail.com; 5 Vascular and Interventional Radiology Department, Cardarelli Hospital, I-80131 Naples, Italy; effecorvino@gmail.com; 6 Radiology Unit, Istituto Diagnostico Varelli, I-80126 Naples, Italy; orlando.catalano@istitutovarelli.it

**Keywords:** ureteral stent, nephrostomy catheter, complications, malpositioning, diagnostic imaging

## Abstract

Ureteral involvement by a tumor is common, and both partial and complete obstructions can result in symptoms that are distressing and debilitating, especially in cancer patients for whom the resection of the primary tumor is not considered an option. Maintaining ureteric patency in these patients is a challenge. In addition, in cases where a patient has undergone nephroureterectomy due to primary transitional cell cancer, it becomes necessary to decompress the urinary tract to preserve the contralateral kidney from irreversible damage. This is possibly due to ureteral stenting, both retrograde and anterograde, and percutaneous nephrostomy (PCN). Since imaging plays an important role in the routine monitoring of stents, their more and more increasing use requires radiologists to be familiar with these devices, their correct position, their potential complications, and their consequences. The aim of this review is to offer a comprehensive review of the imaging features of some urinary stents and to show the complications encountered in cancer patients as a direct consequence of an invasive diagnostic or therapeutic procedure. Specifically, we focus on ureteral stents and PCN.

## 1. Introduction

Ureteral obstructions are one of the most common clinical problems, with etiologies that range from calculi to strictures secondary to surgery or radiation therapy [1]. The increasing use of urinary devices in the management of urinary tract diseases requires radiologists to be familiar with them. As a matter of fact, diagnostic imaging plays an important role in the routine monitoring of stent function and in the evaluation of the consequences and complications of incorrect positioning [2]. This is particularly relevant for cancer patients with a primary or secondary ureteric obstruction who need ureteral stenting. In many cases, the palliative nature of this procedure makes the assessment of associated complications important in terms of determining the success of urinary diversion [3]. Urinary tract decompression is particularly important in order to rescue the kidneys from irreversible damage caused by a secondary malignant ureteric stricture or benign compression. A good knowledge of urinary devices is especially important for those cancer patients who have already undergone nephroureterectomy for a primary urinary tract cancer or have a bilateral obstruction, or with a previous history of renal atrophy on one side and a current obstruction on the other side [4].

Even though some patients do not experience any side effects or complications, others have multiple stent-related ones. Despite their use being careful and correct, indeed, complications may inevitably arise. Some complications are inherent to stent placement and others come from stent design or the material used and the applied coating. It is important for a radiologist to recognize iatrogenic complications and distinguish them from normal post-procedural findings, avoiding all potential interpretative pitfalls [3,4].

The clinical picture does not reliably allow the early detection of complications as they may be subtle and slowly developing, whereas radiology plays an important role in early detection [1]. Some injuries heal spontaneously, while others require adequate intervention. Iatrogenic changes may be detected by different imaging techniques, including unenhanced and contrast-enhanced urinary tract X-rays (urography, cystography, ascending pyelography, and trans-nephrostomy pyelography), angiography, trans-abdominal or trans-rectal sonography (US) (including contrast-enhanced US, although microbubbles are not excreted by the urinary tract; and color and power Doppler imaging), computed tomography (CT) (including CT-urography and CT-cystography), magnetic resonance imaging (MRI) (including MR-urography), and nuclear medicine [2,3].

The purpose of this article is to offer a comprehensive review of the typical imaging findings of some urinary devices and to illustrate the complications encountered in cancer patients as a direct consequence of diagnostic or therapeutic procedures, with special reference to percutaneous nephrostomy (PCN) and ureteral stenting.

## 2. Generalities on Urinary Devices

Ureteral stents are crucial for managing obstructions and promoting urine flow. They address issues, like ureteral dehiscence and obstructive calculi, and aid in fistula healing. Stents can be retrogradely inserted via cystoscopy or anterogradely percutaneously, offering external drainage or a portal for minimally invasive procedures [4,5]. Antegrade placement is more successful in malignant ureteral obstruction, while retrograde failure is associated with factors like age, cancer with a ureteral orifice invasion, and an extrinsic ureteral obstruction [6,7].

An optimal stent should allow straightforward insertion, be tissue-compatible, and prevent inflammation or blockage. It should be durable, resist encrustation, and have an extended lifespan. Various ureteral stents, including single-J, double-J, and multi-length variations, exist. The prevalent dual-J design prevents migration, and strategically placed perforations enhance drainage [8,9].

Stents come in various materials. Polyethylene rigid stents have been replaced by the more rupture-resistant polyurethane, and copolymers with improved properties offer resistance to encrustation. Metal ureteric stents provide increased flexibility, improved patency rates, and longer palliative relief compared to plastic stents, which require more frequent changes due to encrustation [10,11].

## 3. Indications in Cancer Patients

Ureteric obstruction by a tumor is common, especially in urological, gynecological, or colorectal malignancies. This can be caused by an extrinsic compression, mural infiltration, or both. Radiation therapy to a primary pelvic tumor can also lead to middle or lower ureteric strictures a result of the ischemic fibrosis of the ureter [12]. The presence of a urinary tract obstruction may be very relevant for patient management. For example, a patient may not be able to undergo chemotherapy just because the blood level of creatinine is too high. In this case, removing the hydronephrosis status becomes a critical need. Another case is the use of nephrotoxic drugs [13]. The ureteral obstruction in a cancer patient may be bilateral, or it may be unilateral in a patient with a history of contralateral nephrectomy or with a previously atrophied kidney, as for untreated hydronephrosis. In these scenarios, resolving the obstruction issue becomes a key point of patient management [12,13].

Divergent opinions persist on the strategy for relieving obstructions in the upper urinary tract. Current management approaches include external drainage through percutaneous nephrostomy (PCN) and internal drainage achieved by placing double-J stents. The invasiveness of PCN exceeds that of a double-J stent insertion, potentially resulting in a higher likelihood of inadvertent tube displacement [14]. The intrusive nature of the procedure and the elevated frequency of tube dislodgement could potentially diminish the overall quality of life for patients. Moreover, some patients may express reluctance in accepting a PCN tube due to the presence of an external collection device. Complications may develop during the placement of the urinary tract device or in the following days [15]. Additionally, since the routine exchange of the catheters and stents is required to prevent encrustation and subsequent sepsis, complications may rise because of this substitution [14,15].

## 4. Imaging Findings

**Figure 1:**
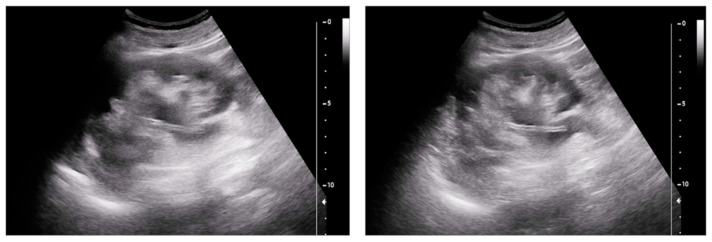
A nephrostomy catheter. On ultrasound, the stent can be readily visualized as a hyper-echoic structure located in the renal pelvis. Note how it is highlighted by the presence of a moderate ureter–pelvis distension.

A plain abdominal X-ray and a CT scan can easily recognize ureteral stents and nephrostomy catheters because of their radio-opaque coating; however, this characteristic can be lost with time. Double-J ureteral stents appear as tubular devices along the ureter with curled ends located in the renal pelvis and urinary bladder; in PCN, the distal pigtail is in the renal pelvis, while the proximal extremity is outside of the abdominal cavity. Previous researchers have documented the numerous advantages of employing dual-energy computed tomography (DECT) to assess ureteral stents. These stents are identified by the DECT scanner and assigned a color value based on their density and chemical composition [16]. When examined using US, the visualization of plastic stents can pose challenges and may not be noticed, while metallic stents exhibit distinct characteristics. Both the upper and lower ends of these stents are easily discernible as hyperechoic structures situated in the renal pelvis and urinary bladder (Figure 1 and Video S1). Furthermore, the inherent high reflectivity of stents generates a comet tail or ring-down artifact. Owing to their innovative design (continuous unfenestrated stents without side or end openings), metal ureteric stents do not generate ureteral jets in the bladder in the same way as plastic stents, which feature a hollow central channel [14,15]. If the stent or catheter extremity is not detectable with convex probes, such as when they have been implanted for a long time and/or there is no adequate distension of the urinary tract to highlight them, it may be useful to use high-frequency linear probes to obtain a better resolution. As a fundamental guideline, it is crucial to know that transducers with higher frequencies offer a superior spatial resolution but at the expense of limited depth penetration. Conversely, transducers with lower frequencies enjoy the benefit of an enhanced depth penetration, albeit at the cost of a diminished spatial resolution [17,18]. In our practical experience, we found it advantageous to use for these purposes the multi-frequency linear probes at the intermediate bandwidth of frequencies ranging between 6 and 9 MHz (commonly used in vascular studies), because they represent a “good compromise” between resolution and depth penetration [18].

## 5. Complications

Although the insertion of both a percutaneous nephro-ureteral stent and retrograde ureteral catheters is safe, complications may occur. The most common complications include suprapubic and flank pain, irritative voiding symptoms, vesicoureteral reflux (VUR), stent malposition, obstruction, encrustation, fracture, knotting, urinary tract infection, cutaneous inflammation at the insertion site, and ureteral erosion or kidney perforation with urinary leakage or bleeding. Each of these complications typically occurs in 1% to 5% of cases [1]. Although not being truly a complication, inadequate obstruction relief should also be considered.

**Figure 2:**
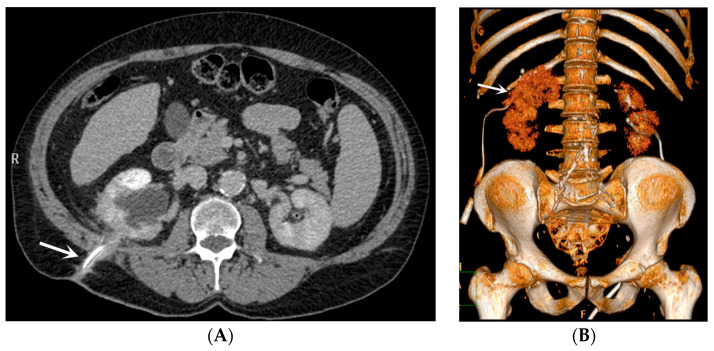
Dislodged right nephrostomy catheter. Axial CT scan (**A**) and volume-rendering CT image (**B**). The device (arrow) is seen in the loin soft tissues. The renal enhancement is heterogeneous and the hydronephrosis persists.

**Figure 3:**
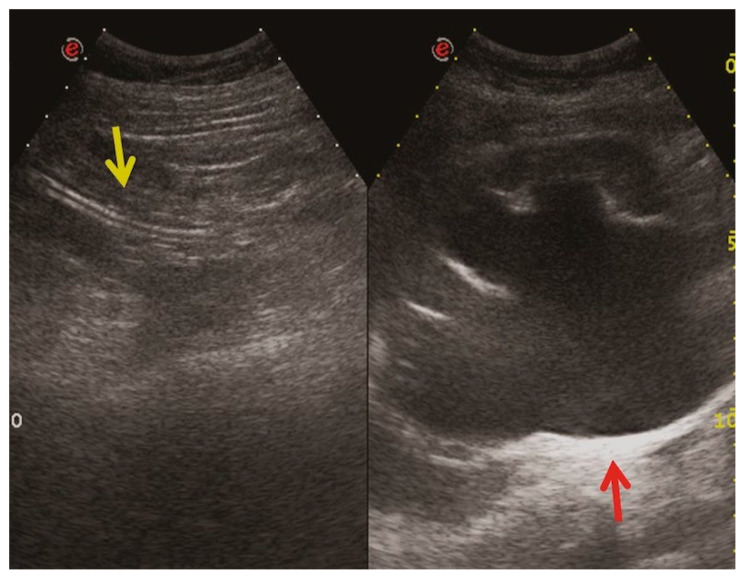
Ultrasound. Nephrostomy catheter seen outside the excretory tract within the retroperitoneal fat (yellow arrows). Marked hydronephrosis persists (red arrow).

**Figure 4:**
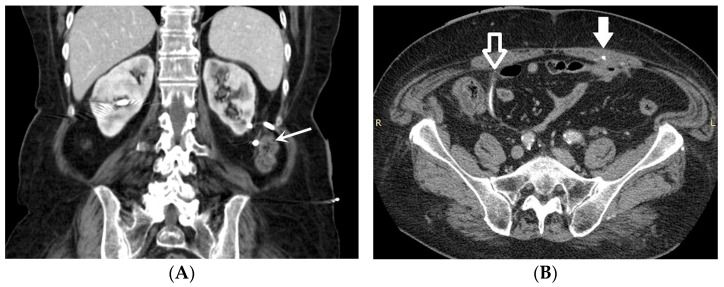
Bilateral nephrostomy. Coronal CT image (**A**) and axial MIP image (**B**). The left-sided catheter is dislodged outside of the urinary tract (arrow).

**Figure 5:**
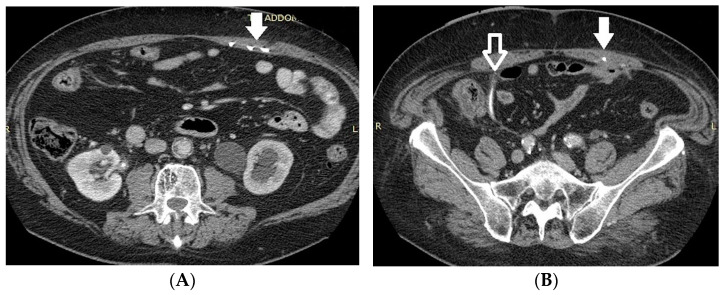
Bilateral splint in a patient with a history of radical cystectomy and ureterocutaneostomy. Axial CT scans. On the left side (**A**), the splint is seen in the abdominal wall (solid arrow), while on the right side (**B**), the splint is seen in place (empty arrow). Left-sided hydronephrosis persists.

**Figure 6:**
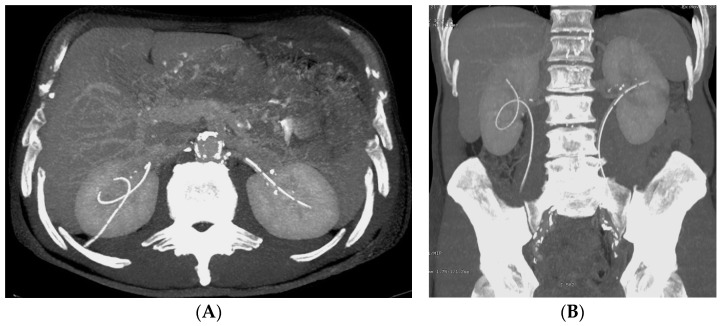
Bilateral splint in a patient with a history of radical cystectomy and ureterocutaneostomy. Axial (**A**) and coronal (**B**) MIP images. On the right side, there is a loop of the splint within the renal pelvis and the tip penetrates the renal parenchyma.

- *Stent discomfort*: Patients with urinary stents frequently experience pain in the suprapubic and flank regions, with an incidence rate of up to 80%. Several factors can contribute to this pain, including vesicoureteral reflux causing an upward surge in intra-ureteral pressure, primarily spasms in the distal ureter, and irritation of the bladder mucosa due to the presence of a foreign body in the bladder [1,2]. It is crucial to emphasize that the root cause of this pain remains unknown to date. Topographically, it corresponds to two distinct regions where patients report discomfort. Approximately 60–77% of patients describe the onset of lateral pain, primarily but not exclusively associated with micturition and vesicoureteral reflux induced by the stent. The occurrence of suprapubic pain, reaching up to 38%, is linked to adverse effects at this level related to the bladder pigtail and the irritation of the bladder trigone [19,20]. Despite the correct placement of contemporary stents, irritative symptoms in the lower urinary tract may manifest in 80–90% of patients. Occasionally, these symptoms can be so unbearable that they necessitate the early removal of the stent. These symptoms, categorized as filling symptoms, emptying symptoms, and post-mictional symptoms, are unequivocally attributed to the irritation of the bladder urothelium by a vesical stent end by causing inflammation and increased activity of the bladder detrusor [21]. Indeed, despite advancements in using biocompatible materials for constructing stents, the epithelium of the renal collecting system, ureter, and bladder reacts to the presence of the foreign entity [2].
- *Vesicoureteral reflux (VUR)*: Anatomically, the ureterovesical junction (UVJ) stands as a crucial structure safeguarding the upper urinary tract from intermittent high pressures in the bladder. Functionally, the UVJ, through its momentary opening, facilitates the passage of urine into the bladder while preventing a retrograde flow into the kidneys during micturition. Several factors contribute to the effective operation of this anti-reflux mechanism: an appropriate length of the intravesical ureter, an oblique angle of ureter insertion into the bladder, and the proper development of the smooth muscle and extracellular matrix capable of compressing the ureteral orifice. Any deviation in these features results in the retrograde flow of urine or VUR. This is inevitable in the presence of an unobstructed stent. Typically, this occurs during the voiding phase when the bladder pressure rises, and the stent, maintaining an open communication between the bladder and the ureter, induces the retrograde flow of urine. In a voiding cystourethrography analysis conducted on patients with stents, reflux during the voiding stage was observed in 80% of cases, likely contributing to the flank pain experienced during voiding by these patients [22].
- *Malposition:* The malposition of a stent is defined as an incorrect position relative to initial placement, while displacement presents a subsequent occurrence in a device that was previously located in the correct position. A stent improperly positioned might assume a sub-pyelic position when the proximal end fails to reach the renal pelvis and a supravesical position when the distal end is detected within the ureter. The origins of this complication predominantly stem from the placement technique, whether it be endoscopy- or fluoroscopy-guided insertion. This underscores the need to verify the accurate positioning of the stent post-placement. Ensuring an adequate length is essential to reduce the occurrence of this complication (Figure 2, Figure 3, Figure 4, Figure 5 and Figure 6) [21,22].

Ideally, stents can be placed under fluoroscopic and/or US guidance, and this technique allows the prompt identification and correction of positioning problems. However, a stent is not a static entity within the urinary tract, and if the patient experiences unusual or persistent symptoms, conventional radiography (plus other imaging technique, as needed) may be necessary [1,2]. A CT scan and abdominal X-ray can quickly recognize malpositioning or displacement or show the coiled extremity of the catheter outside of either the renal collecting system or the bladder. Also, US is useful to demonstrate the incorrect positioning of both double-J ureteral stents and PCN catheters in the renal collecting system. The recognition of a misplacement or displacement is relevant because of the secondary complications that may develop and because the hydronephrosis may persist or start again. If identified early, the incorrectly placed device can be removed or can be placed in the correct position to prevent further undesired effects [22].

**Figure 7:**
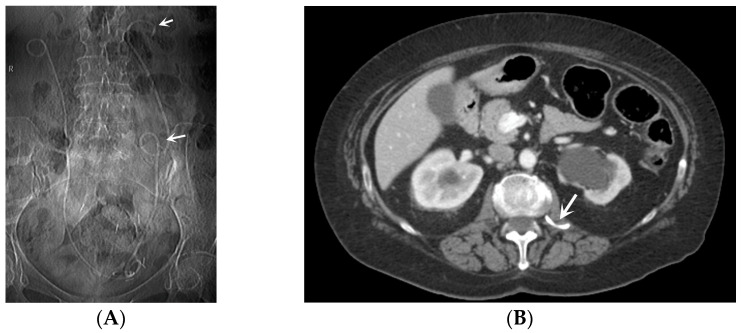
Bilateral stenting. CT scout-view (**A**) and axial CT scan (**B**). Knotted ureteral stent seen perforating the left iliac ureter and reaching the psoas muscle (arrows). Left hydronephrosis persists. The right stent is placed correctly.

**Figure 8:**
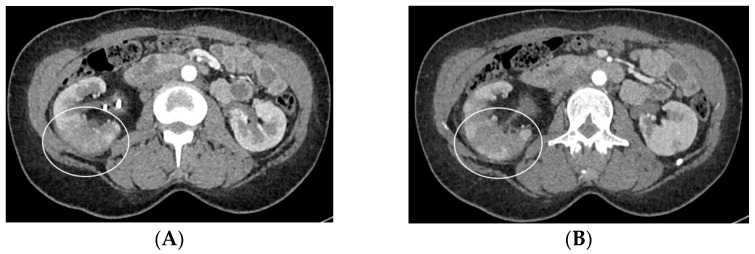

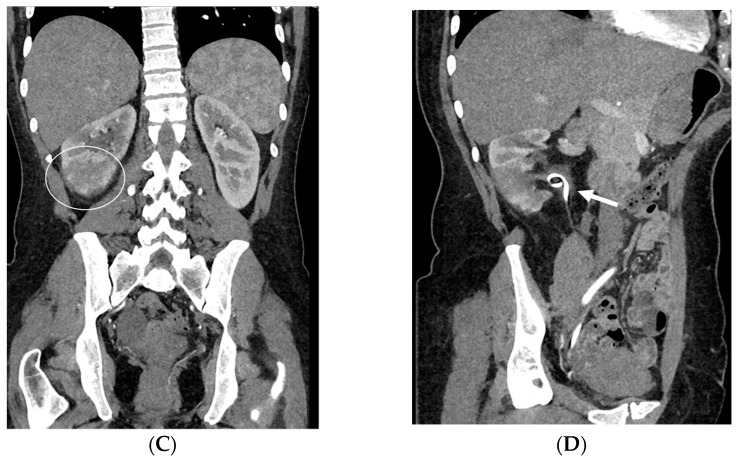
Acute pyelonephritis after stenting procedure. CT portal-phase images in the axial (**A**,**B**), coronal (**C**), and curved MPR (**D**) views demonstrate multifocal regions of diminished enhancement in the medio-inferior region of the right kidney when compared to normal portions of the kidney and a contralateral one (circle). The swirl of the stent is evident in the pelvis (arrow).

- *Obstruction of the stent lumen:* It is a potential occurrence after insertion into the urinary tract. Luminal blockage may arise from hematuria related to the technique or from elevated urine viscosity and debris associated with insertion in an infected system. Clinical manifestations of flank pain can indicate the potential for a stent to be obstructed, but they can also be attributed to a stent that is functioning properly. The evaluation of renal function through blood studies may not necessarily reveal an acute obstruction, particularly if the obstruction is unilateral. Indeed, it is widely recognized that, in patients with a long-standing upper tract obstruction, the placement of a stent may not completely restore the renal collecting system to a normal appearance. During voiding cystography, it is common for most patent stents to exhibit reflux, and this can serve as an indicator of patency. Color Doppler US has also been utilized to assess stent jets [2,3].
- *Encrustation:* Encrustations may develop in the case of long-standing catheters, when the need for obstruction relief is necessary for a long time or when the stent has just been “forgotten”, as for patients lost to follow-up. Encrustation most frequently develops in the endovesical part of a double-J catheter. It is important to identify the presence and extent of the encrustation in order to plan treatment strategies [23,24].
- *Stent fracture*: Due to the “hostile” environment of human urine, a stent fracture can occur. Over time, polyethylene was eliminated as a material because stents made of it turned brittle and fractured in relatively short indwelling times. Stent fractures have also been documented with newer materials. Interestingly, fenestration sites are where most fractures occur. Encrustation probably contributes to stent fragmentation, and the prevalence of both complications is directly proportional to the residence period [2,25,26].
- *Stent Knotting*: It is a rare complication. Most of these knots involve the proximal end of the stent near the coil, but every portion can be affected [27,28]. Previous reports have attributed knot formation to the excessive length of the stent, stent shape (double-J or multicoil), and flexibility or anatomical abnormalities, such as cystocele and ileal conduits. An abdominal X-ray and, especially, a CT scan are more sensitive than other imaging modalities in identifying a ruptured or knotted stent as well as its migration (Figure 7) [29,30].
- *Urinary tract infection*: Stent colonization by bacteria, with an overall incidence ranging from 42% to 90%, is a significant clinical challenge that can lead to a urinary tract infection. In some instances, this infection can result in complications, such as acute pyelonephritis (Figure 8) and renal failure [31,32]. For most patients experiencing a ureteral obstruction, stent placement is carried out with antibiotic prophylaxis, typically administered as a single dose concurrent with the procedure. In cases where a urinary tract infection is already known, the insertion of the stent should be delayed whenever possible until the appropriate treatment with culture-specific antibiotics allows for urine sterilization [32]. US serves as the first-line diagnostic tool to assess the urinary tract in patients presenting with the symptoms of pyelonephritis. Unfortunately, pyelonephritis lacks clear gray-scale findings useful during characterization [33]. Consequently, most patients with clinically suspected pyelonephritis have negative results from US. In cases where imaging is deemed necessary, CT emerges as the preferred modality, providing comprehensive anatomical and physiological information and accurately delineating both intra- and extra-renal pathological conditions. The presence of urinary tract gas, calculi, hemorrhage, renal enlargement, inflammatory masses, and obstruction can be easily detected by CT. Specifically, the affected regions may show a lower attenuation due to edema with pockets of higher attenuation representing the foci of hemorrhage. However, these findings are frequently absent, and unenhanced CT images may appear normal. It is only after the administration of contrast material that the diagnostic features of pyelonephritis become evident. In advanced stages, sepsis is a potential complication, occasionally presenting a critical issue in a debilitated cancer patient. The close monitoring of the patients after the procedure is imperative, with a heightened awareness of the potential for sepsis. The incidence of sepsis following catheter insertion varies between 1.5% and 7%, particularly in patients with pyelonephritis [1,2].

Hence, CT should be performed with precontrast imaging before the administration of the contrast material, followed by postcontrast imaging at approximately 50–90 s after injection, with delayed imaging only if a urinary tract obstruction is suspected. This CT protocol is designed to maximize the information of each phase of study, particularly the nephrographic phase, during which the normal kidney exhibits homogeneous enhancement. Following the administration of the contrast material, nephritis typically manifests as one or more wedge-shaped areas or streaky zones of reduced enhancement extending from the papilla to the renal cortex (Figure 8). Physiopathologically, this finding has been related to the underlying pathophysiology of tubular obstructions caused by inflammatory debris within the lumen, interstitial edema, and vasospasm [34].

**Figure 9:**
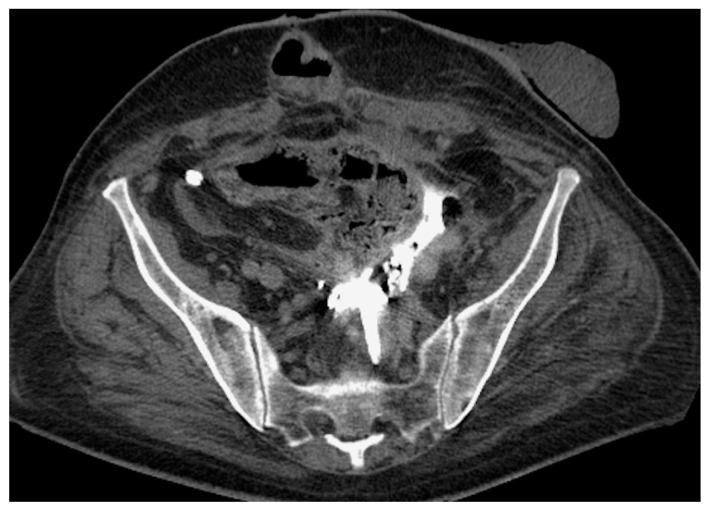
Urinoma in a patient with a perforated ureteral stent. The collection fills with the excreted contrast medium.

- *Urine leakage*: Stents crafted from more rigid materials have the potential to perforate the ureter, collecting system, and kidney parenchyma during placement, leading to the formation of fluid collections or urine leakage, often resulting in a urinoma [35] (Figure 9). A urinoma is a collection of extravasated urine outside of the urinary tract. The optimal diagnostic imaging studies for this condition include contrast material-enhanced CT with delayed imaging (10–20 min), CT–cystography, and a retrograde urethrography [36,37]. In a CT scan, a urinoma may manifest as a restricted or unrestrained collection within the intra- or retro-peritoneal compartment, with the latter occurrence being more prevalent. Its attenuation values can vary from 0 to 20 HUs before the administration of intravenous contrast, subsequently intensifying up to 200 HUs after contrast administration (Figure 9). The irritation from the urine may lead to the formation of a fibrous capsule surrounding the urinoma, which can occasionally become calcified. Not infrequently, a dystrophic calcification of the urinoma may occur, which results from an inflammatory and fibrotic reaction to the extravasated urine [38]. Urinomas, typically small initially, are often resolved on their own without intervention. However, in cases of significant injury or a larger urinoma failing to reabsorb, urological or interventional radiological procedures may be necessary. Neglecting intervention can lead to complications, such as abscess, electrolyte imbalance, hydronephrosis, and urosepsis [37,38].

The primary treatment involves using a drainage catheter and empiric antibiotics, but if ineffective, a percutaneous nephrostomy tube may be inserted, often with a ureteral stent. Surgical reconstruction is reserved for severe cases. Early awareness and prompt treatment are crucial in avoiding more aggressive measures [38].

**Figure 10:**
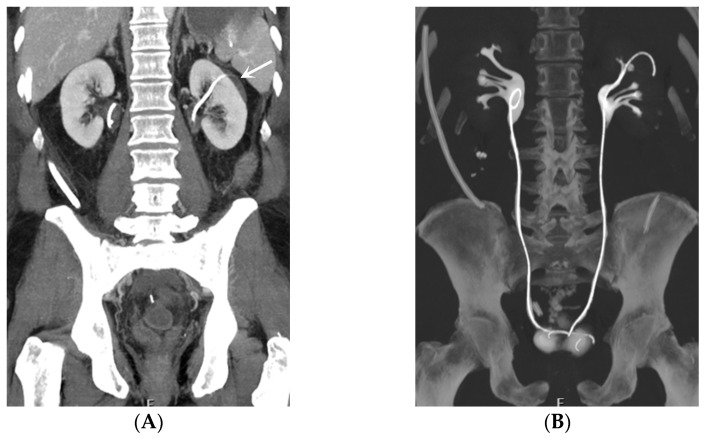
Bilateral stenting of the ureter. Coronal CT image (**A**) and coronal MIP image (**B**). On the left side, the device penetrates the renal parenchyma, causing the formation of a thin subcapsular collection (arrow).

**Figure 11:**
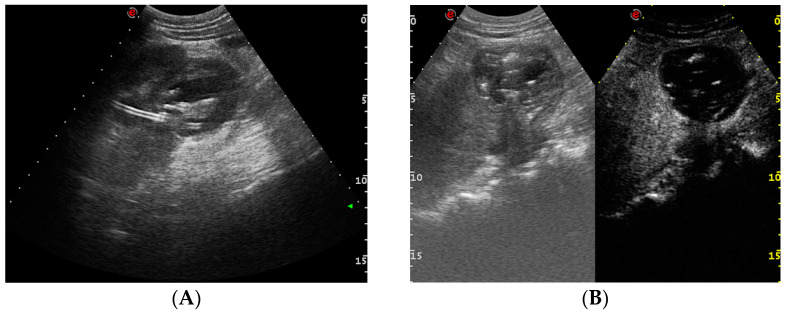
Renal hematoma after the attempted placement of a nephrostomy catheter. US (**A**) and contrast-enhanced US (**B**) showing a hypoechoic, inhomogeneous, non-enhanced collection at the lower renal end.

**Figure 12:**
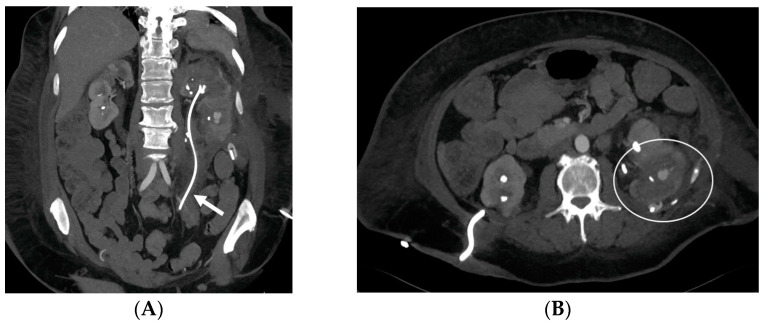
Vascular complication after double-J stent placement (arrow). CT arterial-phase images in the coronal (**A**) and axial (**B**) views demonstrate the presence of a pseudoaneurysm (circle) confined within a perirenal hematoma.

**Figure 13:**
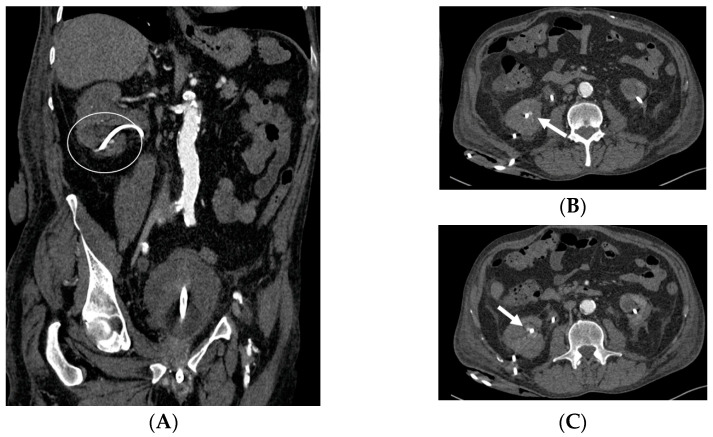
Vascular complication after nephrostomy placement. CT arterial-phase images in the coronal (**A**) and axial (**B**,**C**) views demonstrate the presence of multiple pseudoaneurysms (arrows) along the nephrostomy passage (circle).

**Figure 14:**
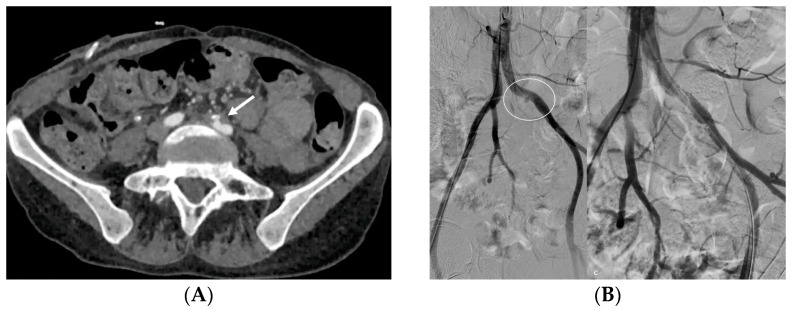
Patient with ureterocutaneostomy after a vesical resection presenting with intermittent bleeding from the cutaneous entry point. The CT study (**A**) demonstrates a pseudoaneurysm of a common iliac artery (arrow), confirmed also by a DSA study (**B**) (circle), in a point closely adjacent to the ureterocutaneostomy. DSA final control image (split image, right) after a covered stenting procedure that confirmed the complete exclusion of the pseudoaneurysm with common iliac artery patency preserved.

- *Bleeding*: The erosion of the stent into the arterial system is a rare and feared complication of ureteral stent placement, which can cause hematomas (Figure 10 and Figure 11), active bleeding, or pseudoaneurysm (Figure 12, Figure 13 and Figure 14). To avoid mortality from these complications, a high level of clinical suspicion is essential. Intermittent hematuria in a patient with a stent is typically the usual clinical scenario. However, massive hematuria and circulatory collapse can occur due to the manipulation of the ureteral stent [2,39].

Diagnosis through clinical examination or any imaging procedure may pose challenges. Angio-CT and angiographic evaluation become necessary, especially when the diagnosis is not initially considered. At a dual-phase CT, a jet or focal area of hyper-attenuation within a hematoma in the initial images that fades into an enlarged hematoma in delayed images is the classic pattern of active extravasation. When there is active extravasation of blood, the contrast-enhanced blood mixes with the fresh and clotted blood that is already present in the hematoma, thus creating high-attenuation shapes resembling a jet or fountain with a tapered edge or spiraling eddy currents with ill-defined edges. In one study, these findings were described as a jet (42% of cases), “diffuse density in hematoma” (37%), and “focal density in hematoma” (21%) [39,40,41].

In contrast to active extravasation, an isolated pseudoaneurysm is contained by connective tissue or the outer layers of vessel wall. This is the reason why the pseudoaneurysm is nearby a vessel, and its size and attenuation do not change even after the contrast material is washed out of the arterial system in delayed images. The outer layers of the vessel wall or connective tissue confine the contrast agent under pressure, shaping it into a round or oval form with a well-defined edge in CT images. Having a precise diagnosis is crucial for therapy, which may encompass open surgical techniques, interventional radiologic approaches, or a combination of both [42,43,44].

## 6. Conclusions

Various medical devices manage urinary tract obstructions and commonly appear in routine imaging. It is crucial to carefully check for their presence during abdominal and pelvic studies. Recognizing their misplacement and potential complications is more straightforward in coronal sections if their presence has been initially identified in axial sections. Overlooking seemingly routine devices while swiftly progressing to assess patient issues is a risk [8]. Understanding the device’s proper positioning and function is essential, holding significance in radiologic examinations. Radiologists must identify malpositions or ruptures promptly, informing the responsible physician, as complications can lead to undesirable, and in some cases, fatal consequences [24]. Physicians performing stent insertions bear the responsibility of obtaining informed consent, whose neglect carries management and potential malpractice implications [2]. A multidisciplinary approach with urology and nephrology specialists proves valuable in addressing complex clinical challenges.

## Data Availability

The data presented in this study are available upon request and are not publicly available due to restrictions regarding patients’ privacy.

## Supplementary Materials

The following supporting information can be downloaded at: https://www.mdpi.com/article/10.3390/medicina60020338/s1. Video S1: Nephrostomy catheter. On the ultrasound clip, the stent can be readily visualized as hyper-echoic structures located in the renal pelvis. Note how it is highlighted by the presence of a moderate ureter–pelvis distension.
